# Effects of Chicken Protein Hydrolysate as a Protein Source to Partially Replace Chicken Meal on Gut Health, Gut Microbial Structure, and Metabolite Composition in Cats

**DOI:** 10.3390/vetsci12040388

**Published:** 2025-04-21

**Authors:** Tong Yu, Fabian Humbert, Dan Li, Arnaud Savarin, Mingrui Zhang, Yingyue Cui, Haotian Wang, Tianyu Dong, Yi Wu

**Affiliations:** 1 State Key Laboratory of Animal Nutrition and Feeding, College of Animal Science and Technology, China Agricultural University, Beijing 100193, China; 2022304010128@cau.edu.cn (T.Y.); zhangmingrui@cau.edu.cn (M.Z.); sy20233040846@cau.edu.cn (Y.C.); wanghaotian@cau.edu.cn (H.W.); dongtianyu@cau.edu.cn (T.D.); 2 Jiangxi Wing Biotechnology Co., Ltd., Shanghai 200001, China; fabian.humbert@symrise.com (F.H.); dan.li@symrise.com (D.L.); arnaud.savarin@symrise.com (A.S.)

**Keywords:** companion animal, gut health, gut microbial community, metabolite profile, protein hydrolysate

## Abstract

Protein hydrolysates positively affect intestinal function in both humans and animals, but their impact on gut health and the gut microbial profile in cats has not been thoroughly investigated. In this study, a total of 30 adult cats were randomly assigned to one of three dietary treatments for a 60-day feeding trial. The three dietary treatments were as follows: (1) basal diet (CON), (2) diet containing 15% powdered chicken protein hydrolysate (HP15%), and (3) diet containing 15% liquid chicken protein hydrolysate (HL15%). Compared to the CON group, the HP15% group had a decreased calprotectin levels and fecal gases emissions (*p* < 0.05). A higher abundance of Bacteroidota, Veillonellaceae, and Bacteroidaceae, while a lower abundance of Firmicutes was showed in the HL15% group than that in the CON group (*p* < 0.05). At the genus level, compared with the CON group, an increased abundance of *Bacteroides* spp. and *Bifidobacterium* spp. was showed, whereas a reduced abundance of *Alloprevotella* spp. was presented in the HP15% and HL15% groups (*p* < 0.05). The metabolomic analysis revealed 1405 distinct metabolites between the HP15% and CON groups (*p* < 0.05, VIP-pred-OPLS-DA > 1), and the level of cholic acid decreased while the level of isodeoxycholic acid increased in the HP15% group (*p* < 0.05). The metabolomic analysis revealed 1910 distinct metabolites between the HL15% and CON groups (*p* < 0.05, VIP-pred-OPLS-DA > 1), and the levels of 4-coumaryl alcohol and enterolactone increased in the HL15% group (*p* < 0.05). In summary, this study suggested that partially replacing chicken meat with chicken protein hydrolysate in the diet of cats helps regulate the gut microbial community and metabolite profile and improves intestinal health.

## Simple Summary

Protein hydrolysates, formed by the enzymatic conversion of proteins into smaller molecular weight, are known to enhance the well-being of mammals and humans. Nevertheless, the influence of protein hydrolysates on feline gut health remains insufficiently explored. The purpose of this research was to investigate the impact of chicken protein hydrolysate as a protein source on the gut microbiota profiles and gut health in cats. The results indicated that partially replacing chicken meat with chicken protein hydrolysate improved the structure of the gut microbiota and metabolite composition and was beneficial to the intestinal health of cats.

## 1. Introduction

The pet population is growing worldwide, and pet food, being an inelastic demand, is expected to continue growing steadily. Pets are regarded as family members, and people attach great importance to pet health. Pet owners are increasingly interested in functional and sustainable ingredient sources that not only meet the nutritional requirements of the pet but also help to reduce the risk of disease, benefit the health and well-being, and extend the life expectancy of their pets [1,2,3]. Therefore, it is important to provide protein ingredients with higher palatability and better functional value, thereby contributing to the nutritional and health status of pets.

In the conventional preparation of pet food, animal proteins, such as beef and poultry meal, have historically constituted the primary source of protein. However, in vitro and in vivo studies have demonstrated that protein hydrolysates contain higher proportions of peptides and free amino acids compared to crude proteins, thereby facilitating digestion and absorption in the small intestine [4,5]. The hydrolysis of animal proteins involves the breakdown of proteins (>10 kDa) into smaller molecules, such as amino acids (75–204 Da), short peptides (100–1000 Da), and denatured proteins through chemical, enzymatic, and thermal processes, yielding products with various biological activities, including immunomodulation, antioxidant effects, and lower allergenicity [6,7,8]. Several studies have investigated the use of protein hydrolysates in pet feed. In a study investigating the effects of feeding domestic cats on wool hydrolysate, an increase in intestinal short-chain fatty acids and an improvement in apparent digestibility were observed [9]. Another study showed that cats consuming a mixture of protein hydrolysate from black soldier fly larvae and schizochytrium had elevated levels of immunoglobulin G and superoxide dismutase, along with reduced concentrations of malondialdehyde [10]. Nevertheless, there is a paucity of systematic studies examining the effects of protein hydrolysates on the health of pets, particularly in cats and their specific organs, such as the gut.

The gastrointestinal tract serves multiple functions in the body. In addition to being the primary site for nutrient digestion and absorption, it also acts as a critical defense mechanism against pathogenic and toxic invasions [11]. Disruptions in gut health can manifest as symptoms such as loss of appetite, diarrhea, constipation, and vomiting, which highlight the direct correlation between gut health and overall well-being. The gut microbiota, which resides within the gastrointestinal tract, constitutes a heterogeneous and equilibrium assemblage of microorganisms [12]. The key functions of the gut microbiota include facilitating food digestion, producing microbial metabolites, and maintaining the integrity of the mucosal barrier [13]. The composition and functionality of the gut microbiota are closely intertwined with the health status of cats. Dysbiosis, a state of microbial imbalance, can lead to reduced bacterial diversity, loss of beneficial bacteria, and overgrowth of pathogens [14]. In cats, dysbiosis is linked to a number of untoward effects, including fecal hetero odor, poor fecal score, and intestinal inflammation [15]. Diet is identified as the principal determinant of the biodiversity and functional attributes of the gut microbiota in mammalian species [16,17]. Therefore, optimizing the composition and structure of feline diets to enhance the architecture of the gut microbiota, thereby improving the intestinal health and overall well-being of cats, is of significant importance.

The aim of this study was to assess the influence of incorporating chicken protein hydrolysate, partially replacing chicken meat ingredients, on the gut inflammation index, fecal odor, gut microbiota composition, and metabolite profiles in an extruded diet, in comparison to conventional chicken meal, for adult cats. This approach aligns with pet owners’ increasing focus on improving their pets’ well-being and offers insights into developing premium cat food formulations that meet modern health-conscious pet care demands. Based on previous research demonstrating the beneficial effects of protein hydrolysates on feline metabolism and immunity, we hypothesize that chicken protein hydrolysate may enhance intestinal health in cats by modulating gut microbiota structure and metabolism.

## 2. Materials and Methods

### 2.1. Animals

The Institutional Animal Care and Use Committee of China Agricultural University approved the experimental protocols and animal treatment (identification code: AW30803202-1-2). All cats were housed at the Pet Feeding Center, Ministry of Agriculture and Rural Affairs Feed Industry Centre, China Agricultural University, and received care in accordance with the National Research Council’s guidelines.

Thirty adult cats (fifteen males and fifteen females), all exceeding one year of age with no more than a two-year age difference between the oldest and youngest individuals, were selected for this study. Before the commencement of the research, a comprehensive medical examination was performed on these cats, which included blood and serum analyses, urinalysis, assessment of appetite, evaluation of body condition, fecal scoring, and parasite screening. None of the cats had immune-mediated diseases, allergies, or other conditions that could lead to chronic gastrointestinal dysfunction, such as liver disease, pancreatic insufficiency, metabolic disorders, parasitic infections or kidney disease. In addition, during the trial period, the cats were not allowed outdoor access and were prevented from contact with unfamiliar personnel to avoid causing them stress. For three months prior to the trial, the cats were not given any function-related medications or diets associated with the test functions, nor were they subjected to any surgical procedures, immunosuppressive drugs, or antibiotics. Additionally, cats that were unable to consume oral feed, had chronic medical conditions, or were nursing or pregnant were excluded from the study. Thirty healthy cats participated fully in the trial.

### 2.2. Experimental Treatments

Thirty cats were randomly assigned to three treatment groups (mean BW 4.58 ± 0.07 kg, mean age 1.47 ± 0.14 years), each consisting of ten replicates. The 30 cats were evenly divided into six rooms. All the male cats were neutered. All cats were free to move around the room. The dietary treatments administered were as follows: (1) basal diet (CON), (2) diet containing 15% powdered chicken protein hydrolysate (HP15%), and (3) diet containing 15% liquid chicken protein hydrolysate (HL15%). The diets were formulated to meet the nutrient recommendations of NRC (2006). The nutritional level and composition of the basal diet are presented in Table 1. The basal diet consisted of commercially available extruded cat food produced by the Pure & Natural Company. The protein hydrolysate was made from chicken raw materials through hydrolysis, high-temperature sterilization, and then spray-dried to create powdered chicken protein hydrolysate, which was ground into a fine powder. The slurry produced immediately following hydrolysis and thermal sterilization was termed liquid chicken protein hydrolysate. HL15% and HP15% were incorporated as partial replacements for chicken meal in the CON diet. The production process of puffed cat food included: raw material crushing, mixing, high-temperature sterilization, extrusion, cutting, drying, and cooling. These cats were fed the same amount of food at 08:30 and 15:30 each day. The cats had access to drinking water at all times. During the experiment, attention was paid to each cat’s mental status, appetite, defecation condition, and other noteworthy abnormalities. The animal rooms were maintained in a clean and well-ventilated state throughout the study period. Prior to the commencement of this investigative project, a thorough clinical evaluation was conducted on a cohort of cats. This evaluation encompassed blood and serum analyses, urinalysis, an assessment of appetite, an evaluation of body condition, a fecal scoring system, and a parasite screening process.

### 2.3. Molecular Weight Range of Polypeptides

The polypeptide molecular weight distribution was analyzed by high-performance liquid chromatography (HPLC; LC-20AD, Shimadzu, Kyoto, Japan) equipped with an ultraviolet detector (SPD-20A, Shimadzu). Separations were carried out on a hydrophilic size-exclusion chromatography column (TSKgel G2000SWxl, 7.8 × 300 mm, 5 μm; Tosoh Bioscience, Shanghai, China) maintained at 25 °C. The mobile phase comprised 0.1 M sodium sulfate and 0.1 M phosphate buffer (PB, pH 6.7), delivered at a flow rate of 1.0 mL/min. Peptide detection was achieved at 220 nm, corresponding to the characteristic absorbance of the peptide bond.

### 2.4. Fecal Sample Collection

The experiment lasted 60 days, with weekly fecal scoring. The day when the cats were fed different types of cat food is considered Day 1, and the day before that, when the baseline values were collected, is considered Day 0. Before the experiment began, all the cats were fed an ordinary cat extruded diet and water. On days 30 and 60, each cat was individually placed in a cage for fresh fecal sample collection, with adequate water and cat food provided inside the cage and then stored at −80 °C for the analysis of gas emissions and fecal calprotectin. Another portion of the freshly collected feces was also frozen at −80 °C for subsequent analysis of gut microbiota composition and non-targeted metabolomic profiles. The sample was ground into powder after freezing and then stored in the refrigerator.

### 2.5. Fecal Scoring

Fecal scoring was conducted by a specifically trained researcher to observe the characteristics of fresh fecal matter. The evaluation of the fecal samples was conducted using a five-point scale, which is explained in the following manner: 5 = watery; liquid that could be poured; 4 = soft and unformed stool; 3 = soft, formed, and damp stool; maintained shape; 2 = hard, formed, and dry stool; maintained firm and soft; 1 = hard and dry spheres; small hard mass.

### 2.6. Fecal Gases

Fecal gases include ammonia and hydrogen sulfide. The concentrations of ammonia and hydrogen sulfide in the feces were measured using the ZG-1 manual gas sampler. First, break the sealed ends of the gas detection tube in the cutting hole at the front end of the sampler, then insert the detection tube into the sealed bags containing fecal samples, aligning it with the gas to be measured. Rotate the handle of the sampler until the red dot on the handle aligns with the white line on the back cover of the sampler. Pull the handle to the 50 mL mark and secure it in place. When the leading edge of the color change in the detection tube ceases to advance, remove the detection tube and record the concentration of the measured gas.

### 2.7. Fecal Calprotectin

The level of calprotectin was evaluated through the utilization of the commercially available MM-67647O2 Feline Calprotectin) ELISA Research Kit 48T following the instructions delineated by the manufacturer (Jiangsu Meimian Industrial Co., Ltd., Yancheng, China).

### 2.8. Fecal Microbiota Sequencing

Total bacterial DNA was isolated from fecal samples utilizing the QIAamp Fast DNA Stool Mini Kit (Qiagen, Germany). The V3-V4 region of the 16S rRNA gene was amplified with the universal primers 338F (5′-ACTCCTACGGGAGGCAGCAG-3′) and 806R (5′-GGACTACHVGGGTWTCTAAT-3′). The resulting amplicons were pooled in equimolar amounts and sequenced on the Illumina MiSeq platform, generating 300 bp paired-end reads. Raw 16S rRNA sequences were evaluated for quality using fast software (version 0.19.6), and sequence splicing was performed with FLASH software (version 1.2.11). The optimized sequences were clustered into operational taxonomic units (OTUs) at a 97% similarity threshold using UPARSE software (version 11). The taxonomic makeup of each specimen was determined across various taxonomic ranks, and substantial distinctions in abundance between groups were detected by means of the Kruskal–Wallis H test. Alpha diversity was measured with Shannon and Simpson indices in Mothur (version 1.30.2), and the variation among different communities, known as beta diversity, was measured using the Bray–Curtis index and depicted by Principal Coordinate Analysis (PCoA).

### 2.9. Fecal Non-Targeted Metabolomics

A fecal sample weighing 50 mg was deposited into a 2 mL centrifuge vial, accompanied by a 6 mm-diameter grinding ball. The specimens were ground with the Wonbio-96c cryogenic tissue mill (Shanghai Wanbo Biotechnology Co., Ltd., Shanghai, China) for six minutes at a temperature of −20 °C and a frequency of 50 Hz, followed by low-temperature ultrasonic extraction for 30 min at 5 °C and 40 kHz. Subsequently, the samples were stored at −20 °C for 30 min, centrifuged for 15 min at 4 °C and 13,000× *g*, and the supernatant was transferred to an injection vial for LC/MS analysis. Non-targeted fecal analysis was performed on a Thermo UHPLC-Q Exactive HF-X system with an ACQUITY HSS T3 column (Waters, Milford, MA, USA) at Majorbio Bio-pharmaceutical Technology Co., Ltd. (Shanghai, China). Progenesis QI software (version 3.0, Waters Corporation, Milford, MA, USA) was used to initially process the raw LC/MS data. Data refinement included the removal of internal standards and erroneous peaks, including column bleed, noise and derivatization reagent peaks, followed by de-redundancy and peak pooling. Metabolites were identified through a thorough search of databases, primarily focusing on the Majorbio Database, Metlin (https://metlin.scripps.edu/) (Accessed on 2 September 2024), and HMDB (http://www.hmdb.ca/) (Accessed on 2 September 2024). The data matrix was submitted for analysis on the Majorbio cloud platform (https://cloud.majorbio.com) (Accessed on 2 September 2024). Principal component analysis (PCA) and orthogonal partial least squares discriminant analysis (OPLS-DA) were performed using the R package “ropls” (Version 1.6.2), along with a 7-cycle interactive validation to evaluate model stability.

### 2.10. Statistical Analysis

Data analysis was performed using IBM SPSS Statistics 26 (Chicago, IL, USA). A two-factor repeated measures design was used to examine the effects of treatment duration on the data, the effects of different treatments on the data, and their interactions; a two-way ANOVA was employed to assess the impact of different rooms within the same treatment on the outcomes, the effects of different treatments on the outcomes, and their interactions. The R tool was utilized for depicting the microbial sequencing results. Bar charts of bacterial communities were generated with the R ggplot2 library, while heatmaps were made using the R vegan package. The Kruskal–Wallis H test was used to assess significant differences in microbiota levels and fecal scores across the groups. For other analyses, one-way ANOVA was conducted, supplemented by Tukey’s multiple comparison test. A *p*-value of <0.05 was considered statistically significant. The data were expressed as the mean ± standard error of the mean (SEM).

## 3. Results

### 3.1. Molecular Weight Range of Polypeptides

The results of peak area percentage of polypeptides with molecular weight >10,000 Da and the number-average molecular weight (*M*_n_), weight-average molecular weight (*M_w_*) measured in chicken meat, chicken protein hydrolysate powder, and chicken protein hydrolysate liquid are presented in Table 2.

### 3.2. Fecal Scoring

The results of the fecal scores of cats under different diet treatments during the experimental period are presented in Table 3. On days 0, 30, and 60, no significant difference was observed in the fecal scores of the three groups (*p* > 0.05).

### 3.3. Fecal Calprotectin

**Figure 1:**
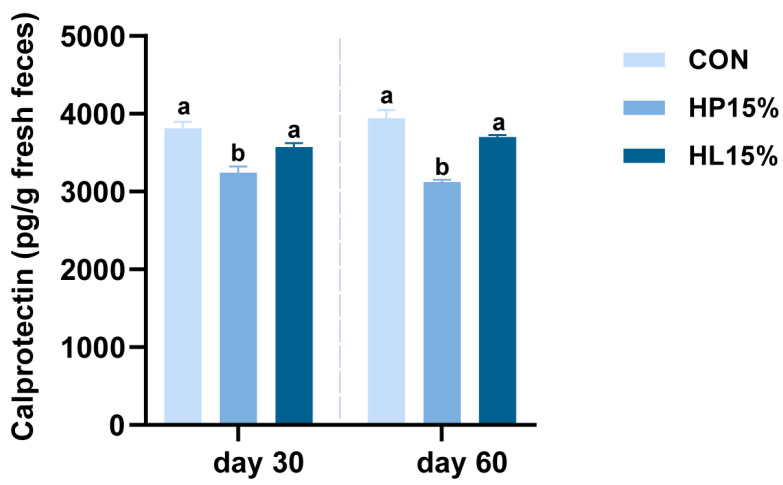
Impact of various dietary regimens on the calprotectin concentration in the feces of cats on Day 30 and Day 60. (1) CON group: basal diet. (2) HP15% group: diet containing 15% powdered chicken protein hydrolysate. (3) HL15% group: diet containing 15% liquid chicken protein hydrolysate. *n* = 10. ^a,b^ Different superscript letters denote a statistically significant difference (*p* < 0.05).

Figure 1 presents the fecal calprotectin levels measured from fresh feces of cats in the different treatments. Shapiro–Wilk tests indicated normal distribution for all groups (Day 30: CON *p* = 0.136, HP15% *p* = 0.661, HL15% *p* = 0.273; Day 60: CON *p* = 0.304, HP15% *p* = 0.864, HL15% *p* = 0.687). Levene’s test confirmed homogeneity of variance (Day 30: *p* = 0.242; Day 60: *p* = 0.509). Repeated measures ANOVA with time as within-subject factor revealed significant temporal effects (*p* < 0.05) but no treatment × time interaction (*p* ≥ 0.05), indicating calprotectin changes were time-dependent but treatment-independent. Two-way ANOVA showed no significant room effects or room × treatment interactions. Compared to CON, HP15% significantly reduced calprotectin levels by 15.05% (Day 30) and 20.77% (Day 60) (both *p <* 0.05). No significant differences were observed between HL15% and CON groups at either timepoint.

### 3.4. Fecal Gases Emissions

**Figure 2:**
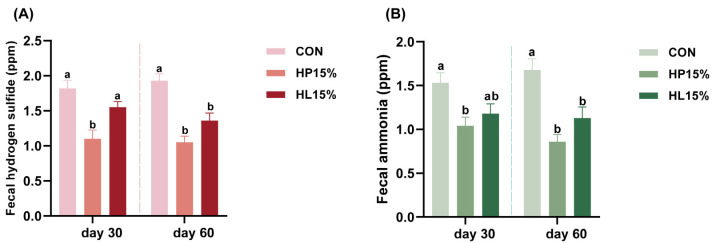
Effects of different diets on fecal hydrogen sulfide and ammonia contents in cats. (**A**) The content of fecal hydrogen sulfide on Day 30 and Day 60. (**B**) The content of fecal ammonia on Day 30 and Day 60. (1) CON group: basal diet. (2) HP15% group: diet containing 15% powdered chicken protein hydrolysate. (3) HL15% group: diet containing 15% liquid chicken protein hydrolysate. *n* = 10. ^a,b^ Different superscript letters denote a statistically significant difference (*p* < 0.05).

Figure 2 illustrates the fecal hydrogen sulfide and ammonia levels measured from fresh feces of cats exposed to the different treatments. Shapiro–Wilk tests indicated normal distribution for all groups (Hydrogen sulfide—Day 30: CON *p* = 0.211, HP15% *p* = 0.276, HL15% *p* = 0.793; Day 60: CON *p* = 0.893, HP15% *p* = 0.345, HL15% *p* = 0.945. Ammonia–Day 30: CON *p* = 0.762, HP15% *p* = 0.238, HL15% *p* = 0.680; Day 60: CON *p* = 0.523, HP15% *p* = 0.704, HL15% *p* = 0.812). Levene’s test confirmed homogeneity of variance (Hydrogen sulfide—Day 30: *p* = 0.950; Day 60: *p* = 0.913. Ammonia—Day 30: *p* = 0.826; Day 60: *p* = 0.313). Repeated measures ANOVA with time as within-subject factor revealed significant temporal effects (*p* < 0.05) but no treatment × time interaction (*p* ≥ 0.05), indicating gas level changes were time-dependent but treatment-independent. Two-way ANOVA showed no significant room effects or room × treatment interactions. Compared to CON, HP15% significantly reduced hydrogen sulfide levels by 39.56% (Day 30) and 45.60% (Day 60), and ammonia levels by 32.03% (Day 30) and 48.81% (Day 60) (*p* < 0.05). No significant differences were observed between HL15% and CON groups on Day 30. On Day 60, HL15% showed 29.53% lower hydrogen sulfide and 32.74% lower ammonia versus CON (*p* < 0.05), with no significant difference in ammonia levels between HL15% and HP15% groups on Day 30.

### 3.5. The Richness and Diversity of Fecal Microbiota

**Figure 3:**
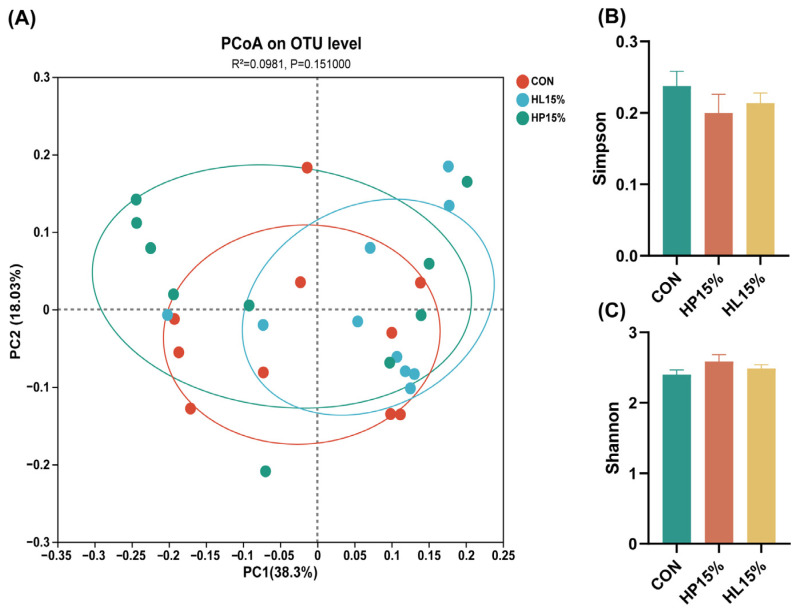
Effects of different diets on the fecal microbial diversity in cats on day 60. (**A**) PCoA at the OTU level. (**B**) Simpson index. (**C**) Shannon index. (1) CON group: basal diet. (2) HP15% group: diet containing 15% powdered chicken protein hydrolysate. (3) HL15% group: diet containing 15% liquid chicken protein hydrolysate. *n* = 10.

At the OTU level, PCoA analysis revealed no clear segregation in fecal microbial community clustering among the three treatment groups (Figure 3A). Similarly, neither the Shannon nor Simpson indices showed significant differences across the groups (Figure 3B,C).

### 3.6. Fecal Microbiota Composition

**Figure 4:**
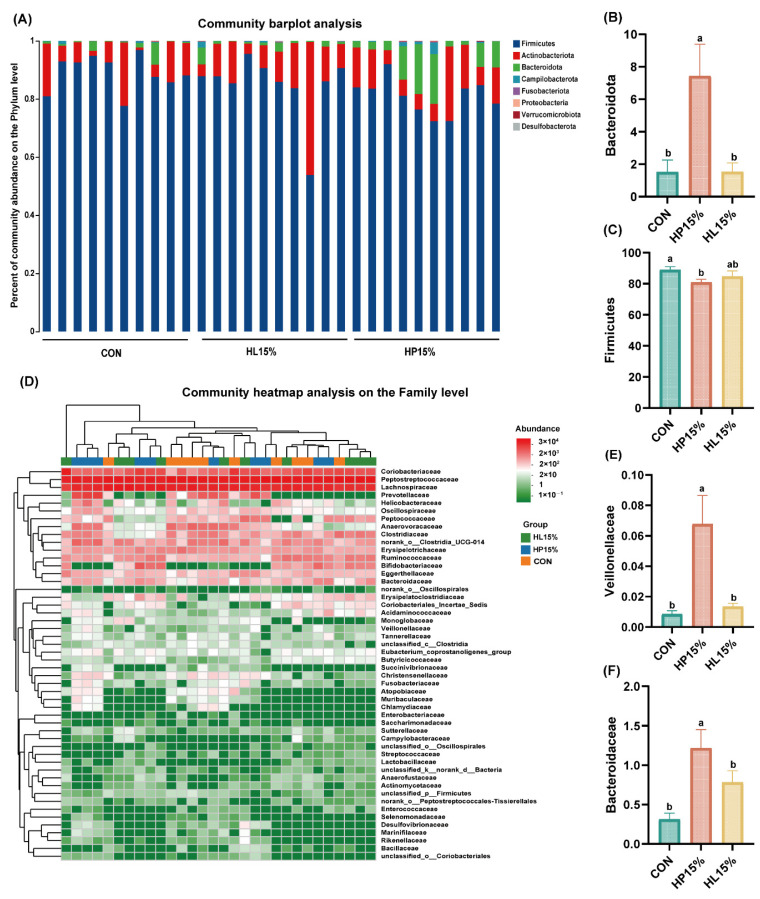
Alterations in the composition of fecal microbiota in cats subjected to various dietary regimens on day 60. (**A**) Relative abundance of fecal microbiota at the phylum level. (**B**) The abundance of Bacteroidota. (**C**) The abundance of Firmicutes. (**D**) The abundance of fecal microbiota at the family level. (**E**) The abundance of Veillonellaceae. (**F**) The abundance of Bacteroidaceae. (1) CON group: basal diet. (2) HP15% group: diet containing 15% powdered chicken protein hydrolysate. (3) HL15% group: diet containing 15% liquid chicken protein hydrolysate. *n* = 10. ^a,b^ Different superscript letters denote a statistically significant difference (*p* < 0.05).

At the phylum level, the abundance of Bacteroidota was higher in the HP15% group than that in the CON and HL15% groups (*p* < 0.05; Figure 4A,B). The abundance of Firmicutes was markedly diminished in the HP15% group relative to the CON group (*p* < 0.05; Figure 4A,C). At the family level, Veillonellaceae and Bacteroidaceae were elevated in cats consuming the HP15% diet compared with those in cats consuming the CON and HL15% diets (*p* < 0.05; Figure 4D–F). However, there was no significant difference in the abundance of Bacteroidota, Firmicutes, Veillonellaceae, and Bacteroidaceae between the HL15% and CON groups.

**Figure 5:**
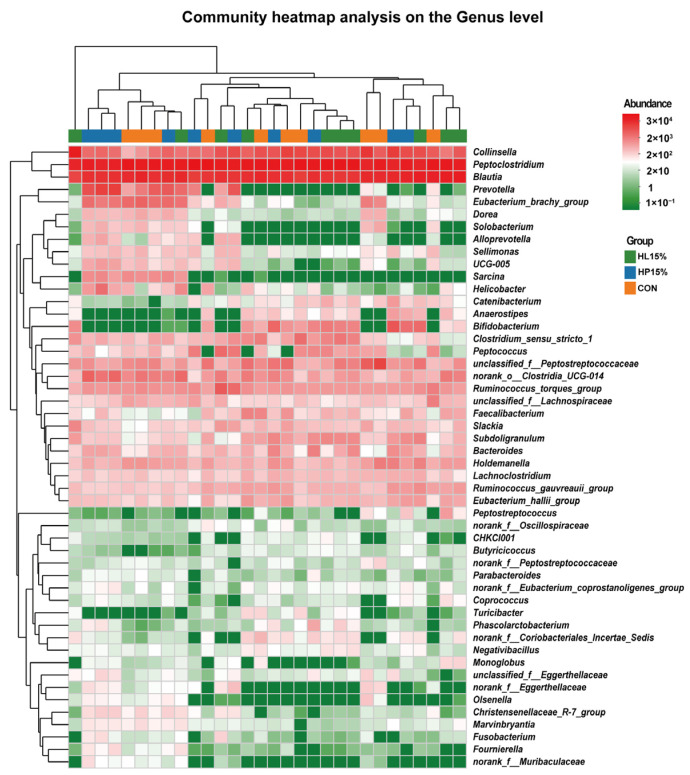
The abundance of fecal microbiota on genus level on day 60. (1) CON group: basal diet. (2) HP15% group: diet containing 15% powdered chicken protein hydrolysate. (3) HL15% group: diet containing 15% liquid chicken protein hydrolysate. *n* = 10.

Figure 5 illustrates the changes in the composition of fecal microbiota at the genus level. Cats fed the HP15% and HL15% diets showed significantly higher abundances of *Bacteroides* spp., *Anaerostipes* spp., and *Bifidobacterium* spp. compared to those fed the CON diet (*p* < 0.05). Conversely, the abundances of *Peptostreptococcus* spp., *norank_f_Peptostreptococcaceae* spp., *UCG-005* spp., *Solobacterium* spp., and *Alloprevotella* spp. were significantly lower in the HP15% and HL15% groups than in the CON group (*p* < 0.05).

### 3.7. Fecal Metabolomic Composition

**Figure 6:**
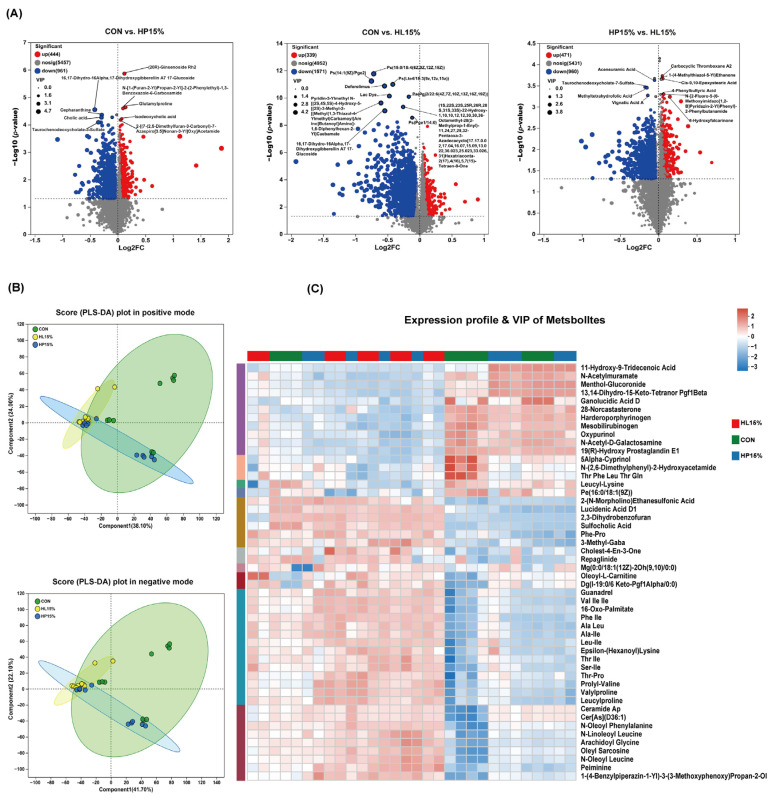
Changes in the metabolites of feces in cats exposed to the different dietary treatments on Day 60. (**A**) Volcano plots presenting differential metabolites in comparisons of the CON group with the HP15% group, the CON group with the HL15% group, and the HP15% group with the HL15% group (VIP-pred-OPLS-DA > 1, *p* < 0.05). (**B**) Scatter plot of PLS-DA results for different metabolites in the CON, HP15%, and HL15% groups in both positive and negative ion modes. (**C**) The assessment of differential metabolites across the three groups was augmented by VIP scoring and a hierarchical clustering heatmap. (1) CON group: basal diet. (2) HP15% group: diet containing 15% powdered chicken protein hydrolysate. (3) HL15% group: diet containing 15% liquid chicken protein hydrolysate. *n* = 10.

Figure 6 presents the differential metabolite analysis results. Between the CON and HP15% groups, we identified 1405 significantly different metabolites (*p* < 0.05, VIP-pred-OPLS-DA > 1), with 444 upregulated and 961 downregulated in the HP15% group. Compared to the CON group, the HL15% group showed 1571 downregulated and 339 upregulated metabolites (*p* < 0.05, VIP-pred-OPLS-DA > 1). When comparing the HP15% and HL15% groups, we detected 1431 differential metabolites, consisting of 960 downregulated and 471 upregulated in the HL15% group (VIP-pred-OPLS-DA > 1, *p* < 0.05). For data analysis, PLS-DA diagrams were applied in both the negative and positive ion modes. The results showed that there was no clear segregation in the metabolite profiles within the three groups (Figure 6B). In comparison to the CON group, the OPLS-DA model’s VIP score revealed that 308 metabolites in the HL15% group exhibited a VIP > 2 (Figure 6C). These included downregulated proctolin (VIP = 4.2241), spinasaponin A (VIP = 3.6862), ginsenoyne B (VIP = 3.5551), amphibine H (VIP = 3.5382), isopropylmaleic acid (VIP = 3.5027), and flocoumafen (VIP = 2.9979). Meanwhile, compared to the CON group, the concentrations of 4-coumaryl alcohol (VIP = 2.8119), (+/−)-Enterolactone (VIP = 2.6261), haloxon (VIP = 2.6137), and ethenoadenosine (VIP = 2.084) exhibited increased levels in the HL15% group. In the HP15% group, the concentrations of ginsenoyne B (VIP = 4.3105), fleroxacin (VIP = 3.7087), ethylmorphine (VIP = 3.5891), cichorioside D (VIP = 3.4368), N-Acetyl-Tryptophan (VIP = 3.2991), glutamylproline (VIP = 3.2927), and isodeoxycholic acid (VIP = 3.2531) were greater than those in the CON group, while the concentrations of 17-Dihydroxygibberellin A7 17-Glucoside (VIP = 2.8968), cepharanthine (VIP = 2.2744), 16,17-Dihydro-16Alpha (VIP = 2.1937), and cholic acid (VIP = 2.1271) were found to be present at reduced levels compared to the CON group.

**Figure 7:**
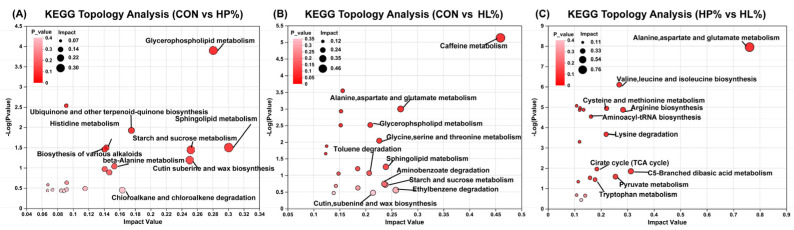
Analysis of KEGG topology regarding differential metabolites on day 60. (**A**) The CON group vs. the HP15% group; (**B**) the CON group vs. the HL15% group; and (**C**) the HP15% group vs. the HL15% group. (1) CON group: basal diet. (2) HP15% group: diet containing 15% powdered chicken protein hydrolysate. (3) HL15% group: diet containing 15% liquid chicken protein hydrolysate. *n* = 10.

Pathway topology analysis was conducted with the curated KEGG pathways serving as the reference framework (Figure 7). With the validated metabolites and their concentration fluctuations, 10 pathways with significant disruptions, characterized by high impact scores (>0.1) and reduced *p*-values, were distinguished between the CON and HP15% groups. These pathways included sucrose metabolism, glycerophospholipid metabolism, histidine metabolism, sphingolipid metabolism, ubiquinone, and other forms of terpenoid-quinone biosynthesis, starch, and biosynthesis of various alkaloids, which were not identified in the HP15% group compared to the HL15% group. Seven disrupted pathways with greater impact scores (>0.1) and lower *p*-values were observed in the comparison between the CON and HL15% groups, including caffeine metabolism, glycine, serine and threonine metabolism, alanine, aspartate and glutamate metabolism, glycerophospholipid metabolism, and histidine metabolism. In the HP15% group, 6 disrupted pathways with greater impact scores (>0.1) and lower *p*-values than the HL15% group were observed, including alanine, aspartate and glutamate metabolism, valine, leucine and isoleucine biosynthesis, cysteine and methionine metabolism, arginine biosynthesis, C5-Branched dibasic acid metabolism, and the citrate cycle (TCA cycle).

### 3.8. Correlation Analysis Between Genus-Level Gut Microbiota and Differential Metabolites

**Figure 8:**
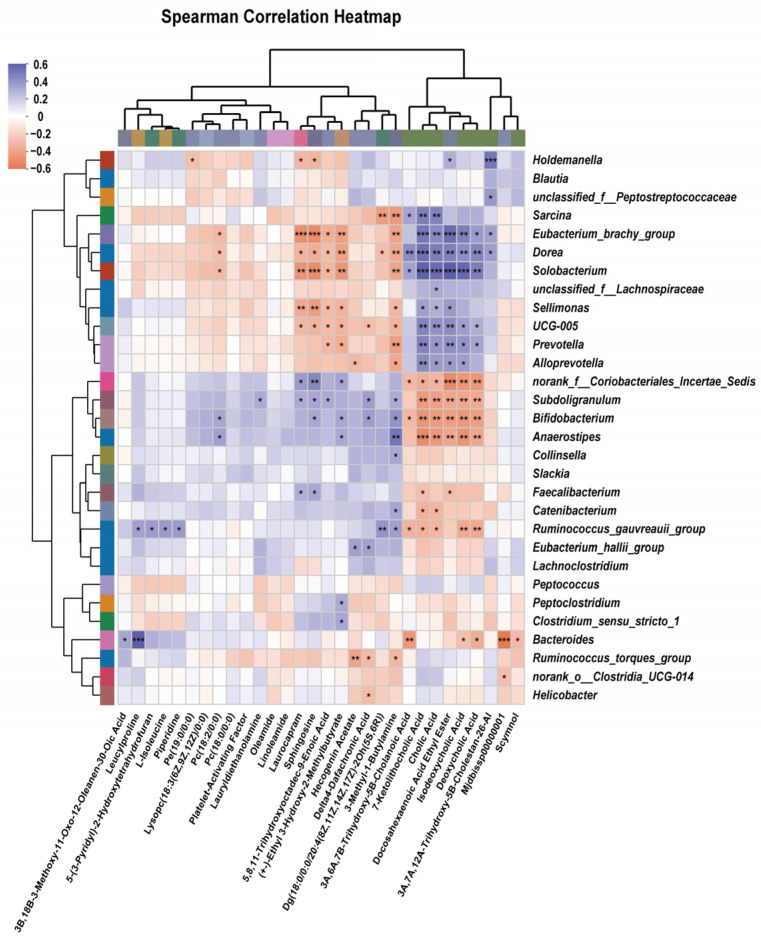
Correlation analysis between the genus-level gut microbiota and differential metabolites on day 60. * *p* < 0.05; ** *p* < 0.01; *** *p* < 0.001. (1) CON group: basal diet. (2) HP15% group: diet containing 15% powdered chicken protein hydrolysate. (3) HL15% group: diet containing 15% liquid chicken protein hydrolysate. *n* = 10.

A Spearman correlation analysis was carried out to assess the connection between the modified metabolites and the changed genera. The top 30 metabolites exhibiting significant associations are shown in Figure 8. The correlation analysis between the intestinal differential metabolites and the microbiome revealed a negative correlation between leucylproline and *Bacteroides* spp. (*p* < 0.05). The abundance of *Bacteroides* spp., *Anaeropstipes* spp., and *Subdoligranulum* spp. was positively correlated with deoxycholic acid (*p* < 0.05). Additionally, the abundance of *Anaeropstipes* spp., *Bifidobacterium* spp., and *Subdoligranulum* spp. was positively correlated with isodeoxycholic acid (*p* < 0.05).

## 4. Discussion

The animal gut harbors a rich microbiota, and the structure of the gut microbiota, along with its metabolism, plays a crucial role in gut health. The method of influencing the gut microbiota through diet to improve animal health has been widely adopted. Recent studies have shown that protein hydrolysates can improve gut health in mice, piglets, and dogs [18,19,20]. However, research on the effects of chicken protein hydrolysate on the gut microbiota and the metabolism of cats is currently limited. Therefore, this study conducted experiments by partially replacing chicken meat with chicken protein hydrolysate powder and liquid in the extruded diet of cats. The results showed that cats fed with protein hydrolysate had significantly reduced fecal calprotectin levels, decreased fecal gases, and altered microbiota composition, with various differential metabolites detected compared to the control group. This was more pronounced in the HP15% group. Notably, the abundance of several beneficial gut bacteria (*Bacteroides* spp. and *Bifidobacterium* spp.) increased in both the HP15% and HL15% groups. In the HP15% group, the levels of primary bile acids decreased while those of secondary bile acids increased. In the HL15% group, the levels of 4-coumaryl alcohol and enterolactone were found to be elevated. These results hold certain positive implications, which will be discussed.

Calprotectin is a cytosolic protein from neutrophils, and its concentration is proportional to the degree of immune-mediated diarrhea and colitis [21]. It has been established that fecal calprotectin serves as a non-invasive biomarker indicative of gut inflammation in dogs [22]. Previous studies have shown that the gut microbiomes of humans, dogs, and cats are similar at the phylum level [23]. In addition, a study showed that adult female dogs fed a natural compound additive with antioxidant properties had improved gastrointestinal health, which was accompanied by a decrease in fecal calprotectin, N-methylhistamine, indole, and cortisol [24]. Notably, gut calprotectin may be involved in the pathogenesis of feline chronic enteropathies, and research has shown that fecal calprotectin concentrations were higher in cats with chronic inflammatory enteropathies or small-cell intestinal lymphoma than those in healthy cats [25]. In this study, cats fed the HP15% diet exhibited lower calprotectin levels in fresh fecal samples at both Day 30 and Day 60 compared to those fed the CON diet. These results suggest that the HP15% diet may help reduce the risk of inflammatory bowel disease in healthy cats. However, these results were not adjusted for fecal dry matter content and require further confirmation through additional evidence.

The production and release of ammonia and hydrogen sulfide can be affected by dietary nutrient levels and the types of ingredients. Intestinal barrier dysfunction and chronic inflammatory gastrointestinal disorders can be triggered by hydrogen sulfide imbalance in the intestinal tract. A finding revealed that the enhancement of bacterial sulfidogenic capacity could result in colonic mucin sulfation and colon epithelial cell viability, which further led to colitis in a murine model [14]. In addition, one study found that irritable bowel syndrome-diarrhea subjects had significantly reduced bacterial diversity and more severe microbiota dysbiosis, which was accompanied by activation in the microbial hydrogen sulfide production pathway [26]. Abnormal rise in fecal ammonia and hydrogen sulfide levels induced by diet can compromise colon epithelial integrity and lead to increased intestinal permeability, resulting in an increased risk for inflammatory bowel disease and colon cancer in animals [27,28]. A study has found that a diet supplemented with casein hydrolysate decreased concentrations of indole, cadaverine, and ammonia, and improved mucosal immunity and barrier function by increasing the protein expressions of immunoglobulin A, mucin-4, and G-protein coupled receptor-43 [29]. In this study, on Day 30, the CON group was found to have significantly higher contents of ammonia and hydrogen sulfide than the HP15% group, and on Day 60, the contents of hydrogen sulfide and ammonia in both the HP15% and HL15% treatments decreased significantly compared with those in the CON treatment. However, studies have shown that the effects of hydrogen sulfide and ammonia in the gut depend on their concentrations, and their reduction may also lead to certain diseases [30,31]. Due to the lack of research on the normal range of hydrogen sulfide in feline intestines, more studies are needed to determine whether the reduction in fecal gases is indeed beneficial for health. The beneficial effects of protein hydrolysate also require further confirmation through subsequent analysis.

Gut microbiota is a significant factor influencing the health status and disease progression of pets [32,33]. The abundant groups in the intestines of cats are Firmicutes, Bacteroidota, Actinobacteria, and Proteobacteria, which is consistent with our findings [34]. In a study, dietary probiotics significantly decreased the abundance of Firmicutes in pet cats and reduced the rate of diarrhea and soft stools [35]. Several studies with cats as target animals have shown that Bacteroidota is one of the dominant bacteria in the intestine, and the abnormal reduction in Bacteroidota abundance has been closely related to intestinal diseases, including inflammatory bowel disease [36,37]. A study on trace elements showed that organic trace elements could increase the abundance of Bacteroidota and improve gut health in cats, which is accompanied by a reduction in inflammation level and the alleviation of gut barrier damage [38]. In this study, the HP15% group exhibited a significant decline in the abundance of Firmicutes and a significant increase in the abundance of Bacteroidota compared to the control group, which suggested that the addition of 15% powdered chicken protein hydrolysate to extruded cat food may have some positive effects, but greater certainty requires further analysis at the genus and family levels. However, the abundance of Firmicutes and Bacteroidota in the HL15% group showed no significant change. This may be due to the lower concentration of protein hydrolysate in the liquid state compared to the powder state.

In our study, the abundance of Veillonellaceae and Bacteroidaceae was higher in cats consuming the HP15% diet than in cats consuming the CON and HL15% diets at the family level. Veillonellaceae can produce rich short-chain fatty acids, such as lactate and propionate [39]. Propionate serves as a substrate for gluconeogenesis in cats [40]. A study has demonstrated that gluconeogenesis from propionate regulates glucose metabolism, thereby suppressing amino acid catabolism, which may confer potential benefits for managing feline insulin resistance and diabetes mellitus [41]. However, since the cats in this study were healthy adults, the applicability of these beneficial effects remains uncertain and requires further investigation for confirmation. A study on the use of prebiotic supplements containing fructooligosaccharides (FOS) and inulin in healthy cats and dogs showed an increase in the abundance of Veillonellaceae [42]. Another study demonstrated that in aged cats, the levels of metabolites linked to harmful processes were found to be reduced, while those connected to beneficial processes were observed to be elevated, with an enhanced presence of Veillonellaceae in the intestine [43]. The impact of the increased abundance of Bacteroidaceae is uncertain. A study has shown that the abundance of Bacteroidaceae in the intestines of healthy cats is significantly higher than in cats with chronic diarrhea [44]. However, due to the limited number of studies related to cats, in other species, the level of intestinal health is inversely related to the abundance of Bacteroidaceae [45,46]. This discrepancy may be attributed to the presence of mechanisms within the feline gut that differ from those in other species, a notion that remains unclear and warrants further investigation. To ascertain whether the structure of the gut microbiota has been ameliorated, further analysis of changes at the genus level is required. As for the genus level, a study has shown that feeding compound probiotics and enzymes can increase the abundance of *Bacteroides* spp. in feces and improve the apparent digestibility of cats, thereby increasing body health and reducing the rate of diarrhea in cats [47]. *Bacteroides* spp. alleviated clinical symptoms of colitis by modulating the regulatory T cells and maintaining the integrity of intestinal barrier, and it was found that the intestinal microbiota of cats with colitis showed a significant decrease in the abundance of *Bacteroides* spp. [48,49]. We observed that *Bifidobacterium* spp. was significantly increased in cats fed the HP15% and HL15% diets compared to those fed the CON diet. *Bifidobacterium* spp. is a beneficial bacterium that contributes to the maintenance of colonic health in animals [50]. In a study, the abundance of *Bifidobacterium* spp. was reduced in the intestines of healthy adult cats fed a high-protein diet, as the high protein level allowed protein-hydrolyzing bacteria to dominate [51]. In our study, the addition of chicken protein hydrolysate in the two experimental groups facilitated the proliferation of *Bifidobacterium* spp. In our research, a remarkable elevation in the abundance of *Bacteroides* spp. and a significant decrease in the abundance of *Bifidobacterium* spp. were shown in the HP15% and HL15% groups. In summary, based on the comprehensive results from the phylum, family, and genus levels, protein hydrolysate has a certain degree of ameliorative effect on the structure and composition of the feline intestinal microbiota.

In recent years, the use of metabolomics to estimate and predict the body’s response to dietary interventions has become a widely used research method [52]. The consumption of nutrients derived from food provides a substrate for microbial metabolism and alters the composition of microbial metabolites in the gut, ultimately affecting the gut and overall health [17]. In our research, an untargeted metabolomic examination of cat feces has been carried out to clarify the impacts of diverse forms of protein hydrolysate on the microbial metabolic pattern in cats. Altogether, 1405 distinct metabolites were detected in the CON and HP15% groups and were significantly upregulated in the HP15% group, such as cholic acid and N-Acetyl-Tryptophan. N-acetyl-tryptophan is an inhibitor of mitochondrial cytochrome c release, and has been reported to exert the ability to protect the nervous system, regulating inflammation, inhibiting macrophage activation, and scavenging free radicals [53,54]. The formation of N-acetyl-tryptophan in the intestine was closely related to the changes in microbial composition. Research has demonstrated that treatment with the probiotic *Bifidobacterium longum* spp. not only elevates levels of N-acetyl tryptophan, tryptophan, butyric acid, and free fatty acids in diarrhea-predominant irritable bowel syndrome patients, but also reveals a positive association through microbiota-metabolite correlation analysis between N-acetyl-tryptophan levels and the abundance of beneficial bacteria, particularly *Bifidobacterium* spp. and *Bacteroides* spp [55,56]. While existing studies demonstrate a positive correlation between N-acetyl-tryptophan and the proliferation of beneficial gut bacteria, emerging evidence has also suggested its association with other pathological conditions, such as Parkinson’s disease [57].

Notably, we observed that when compared to the CON group, the primary bile acid cholic acid content was reduced, while the secondary bile acid isodeoxycholic acid content was increased in the HP15% group. In the classical pathway, cholesterol is metabolized to 7α-hydroxycholesterol by the rate-limiting enzyme cholesterol 7α-hydroxylase, which is then metabolized to cholic acid by sterol 12α-hydroxylase. Studies have shown that the overexpression of cholic acid can aggravate the phenotype of intestinal inflammation [58]. Cholic acid is involved in the regulation of intestinal tight-junction structure and barrier function pathways, and its excessive increase has been shown to inhibit intestinal stem cell renewal and regeneration after acute injury [59]. Research has established that the feces of cats suffering from chronic enteropathy show abnormally elevated levels of primary bile acids compared to those in healthy cats [60]. A study found that diet-induced reduction in cholic acid could promote secondary bile acid metabolism and then activate intestinal bile acid receptors, thereby alleviating high-fat diet-induced gut barrier dysfunction, improving colon tissue damage, and increasing the expression levels of gut barrier proteins zonula occludens-1 and mucin-2 [61]. This evidence supported that the diet containing 15% powdered chicken protein hydrolysate may optimize intestinal health by improving bile acid metabolism, reducing primary bile acid production, and increasing secondary bile acid levels.

Among the differential metabolites between CON and HL15%, the concentrations of some have significantly increased, such as 4-coumaryl alcohol and enterolactone. It is reported that 4-coumaryl alcohol is a flavonoid that has antioxidant and bioactive effects [62]. A review indicated that dietary lignans could be converted into enterolactones by the gut microbiota, and exerted anti-inflammatory and antioxidant effects by regulating the expression of related genes and enzyme activities [63]. In addition, research found that enterolactone was the metabolite of lignan secoisolariciresinol diglucoside from flaxseed and could reduce oxidative stress and inhibit inflammation in vivo [64]. However, high levels of enterolactone are strongly associated with reduced risks of coronary artery disease and cancer [65,66]. Therefore, whether these results indicate definitive positive effects remains unclear and requires further detailed research.

Through KEGG analysis, we found that glycerophospholipid metabolism, sphingolipid metabolism, ubiquinone and other terpenoid-quinone biosynthesis, histidine metabolism, starch and sucrose metabolism, and the biosynthesis of various alkaloids pathways significantly changed in the HP15% group, while glycine, serine and threonine metabolism, alanine, aspartate and glutamate metabolism, glycerophospholipid metabolism, and histidine metabolism pathways significantly altered in the HL15% group. Glycerophospholipid and sphingolipid metabolism are part of lipid metabolism and are related to regulating biological rhythms and absorption of nutrients [67,68]. The synthesis of hydrocarbons and terpenoid derivatives, including oligomers such as vitamins A, E, and K, farnesol, and squalene, which are essential for life activities, is linked to the synthesis of ubiquinone and other terpenoid-quinone pathways [69]. Starch and sucrose metabolism can affect energy utilization, biosynthesis of various alkaloids, and neurotransmission function [70,71,72]. Amino acid metabolism has the capacity to regulate immune cell function and control intestinal inflammation [73,74]. The results of the combined analysis indicate that 30 metabolites, including scymnol, deoxycholic acid, docosahexaenoic acid ethyl ester, cholic acid, 7-ketolithocholic acid, and leucylproline, were significantly correlated with specific microbial genera in this study, which suggests that changes in the gut microbial composition induced by different forms of protein hydrolysate may have a direct impact on the metabolite profile in cats, and highlights the significance of improved gut microbiota in facilitating metabolic performance and general well-being.

In this study, partially replacing chicken meal in the CON group with chicken protein hydrolysate led to improvements in various indicators discussed earlier. We speculate that these improvements may be attributed to the smaller molecular weight of peptides in protein hydrolysate compared to chicken meal, making them more easily digestible and absorbable. Table 2 presents the proportion of polypeptides with molecular weights exceeding 10,000 Daltons in the three raw materials. The HP contained only 3.4%, while the HL had 10.21%, both significantly lower than the 47.37% found in the original chicken material. Polypeptides above 10,000 Daltons are generally considered intact proteins, indicating that HP underwent more thorough hydrolysis than HL. This may explain why the HP15% group showed more significant improvements in fecal parameters, gut microbiota, and metabolic changes compared to the HL15% group. From the processing perspective, HP undergoes an additional spray-drying step compared to HL. One study suggests that spray-drying reduces protein content due to thermal degradation of heat-sensitive amino acids [75]. Furthermore, the breakdown of peptide chains during spray-drying further decreases protein content. Therefore, for pet food manufacturers aiming to maximize the intestinal health benefits of protein hydrolysate in cat diets, it is recommended to apply spray-drying after protein hydrolysis.

There are indeed certain limitations in this study. In the analysis of fecal calprotectin, we did not measure the dry matter content of the stool beforehand. Therefore, while the measured values across groups indicate that chicken protein hydrolysate helps reduce fecal calprotectin levels, the exact magnitude of the concentration decrease may not be entirely precise. Regarding the discussion on metabolites, it remains unclear whether the observed changes (which are concentration-dependent) confer definitive positive effects, as more precise quantitative analyses of these metabolites are required for confirmation. In this study, the chicken protein hydrolysate used in both experimental groups was derived from residual meat on chicken skeletons. In practical applications, using higher-quality chicken meat might yield better results. However, since the hydrolysis process incurs additional production costs, utilizing lower-cost residual skeleton meat offers greater economic feasibility, making the approach more viable for widespread adoption. Additionally, the chicken protein hydrolysate production process requires strict control of hydrolysis conditions. Manufacturers must precisely regulate the procedures to ensure optimal results.

## 5. Conclusions

In this study, analysis of fecal calprotectin levels, fecal gas content, and the fecal microbiome revealed an increase in beneficial bacteria such as *Veillonellaceae*, *Bifidobacterium* spp., and *Bacteroides* spp. Additionally, changes in fecal metabolites, including cholic acids, isodeoxycholic acid, 4-coumaryl alcohol, and enterolactone, were observed in experimental cats. These findings suggest that the diet containing chicken protein hydrolysate as a partial replacement for chicken meat ingredients may modulate gut microbiota composition and metabolic profile, thereby benefiting feline intestinal health and reducing fecal gas emissions. These results suggested that protein hydrolysate in pet food is beneficial in promoting the gut health and well-being of cats.

## Figures and Tables

**Table 1:** Ingredients and nutrient levels in different diets for cats.

Items	CON Group	HP15%	HL15%
Ingredient, %			
Chicken meal	22.86	10.93	9.94
Chicken hydrolyzed liquid	—	—	15.00
Chicken hydrolyzed powder	—	15.00	—
Duck meal	15.90	15.90	15.90
Rice	21.86	20.87	21.86
Peeled pea	19.87	19.87	19.87
Premix ^1^	6.59	6.59	6.59
Flavor enhancers ^2^	2.98	2.98	2.98
Chicken fat	9.94	7.95	7.95
Total	100	100	100
Nutrient level, %			
Dry matter	91.62	91.79	91.24
Crude fat	15.57	16.37	15.68
Crude ash	4.82	4.42	4.39
Crude protein	37.55	37.40	37.09
Crude fiber	2.67	2.60	2.63

Note: ^1^ Premix provided the following per kilogram of feed: vitamin A (14,500 IU), vitamin D_3_ (1000 IU), vitamin E (156 IU), and vitamin B_1_ (32.0 mg), vitamin B_2_ (30.0 mg), vitamin B_3_ (120 mg), vitamin B_5_ (88.0 mg), vitamin B_6_ (13.0 mg), vitamin B_12_ (0.20 mg), FeSO_4_ (100 mg), CuSO_4_ (7.00 mg), CoSO_4_ (1.00 mg), CaI_2_ (20.0 mg), MnSO_4_ (20.0 mg), ZnSO_4_ (68.0 mg), and Na_2_SeO_3_ (0.50 mg). ^2^ The primary component of the flavor enhancers is chicken liver.

**Table 2:** Peak area %, number-average molecular weight (*M*_n_), and weight-average molecular weight (*M*_w_) of >10 kDa polypeptides in different diets.

Groups	>10 kda (%)	*M*_n_	*M*_w_
Chicken meal	47.37	16,748	17,605
HP	3.04	14,079	15,056
HL	10.21	14,207	14,997

Note: (1) Chicken meal: non-hydrolyzed chicken raw material. (2) HP: powdered chicken protein hydrolysate. (3) HL: liquid chicken protein hydrolysate.

**Table 3:** Fecal scores of cats in different diet treatments during the experimental period.

Sampling Date	CON	HP15%	HL15%	SEM	*p*-Value
Day 0	2.55	2.30	2.35	0.13	0.63
Day 30	2.70	2.40	2.35	0.10	0.39
Day 60	2.70	2.55	2.20	0.12	0.26

Note: (1) CON group: basal diet. (2) HP15% group: diet containing 15% powdered chicken protein hydrolysate. (3) HL15% group: diet containing 15% liquid chicken protein hydrolysate. Values are means of each treatment group with their pooled standard error of the means (SEM), *n* = 10.

## Data Availability

Data are contained within the article.
